# Effectiveness of Ocrelizumab in Primary Progressive Multiple Sclerosis: a Multicenter, Retrospective, Real-world Study (OPPORTUNITY)

**DOI:** 10.1007/s13311-023-01415-y

**Published:** 2023-08-23

**Authors:** Clara G. Chisari, Assunta Bianco, Vincenzo Brescia Morra, Massimiliano Calabrese, Fioravante Capone, Paola Cavalla, Carlotta Chiavazza, Cristoforo Comi, Maura Danni, Massimo Filippi, Pietro Iaffaldano, Roberta Lanzillo, Salvatore Lo Fermo, Alessandra Lucisano, Alessandra Lugaresi, Giacomo Lus, Gerolama Alessandra Marfia, Fabiana Marinelli, Massimiliano Mirabella, Lucia Moiola, Chiara Perin, Sabrina Realmuto, Simona Toscano, Maria Trojano, Domizia Vecchio, Francesco Patti, Italian MS Registry

**Affiliations:** 1 Department of Medical and Surgical Sciences and Advanced Technologies “G.F. Ingrassia, ” University of Catania, Via S. Sofia 78, 95100 Catania, Italy; 2 Multiple Sclerosis Center, Fondazione Policlinico Universitario “Agostino Gemelli” IRCCS, Rome, Italy; 3 Multiple Sclerosis Clinical Care, Department of Neurosciences and Reproductive and Odontostomatological Sciences, University “Federico II”, Naples, Italy; 4 Neurology Section of Department of Neuroscience, Biomedicine and Movement, University of Verona, Verona, Italy; 5 Unit of Neurology, Department of Medicine, Neurophysiology, and Neurobiology, University Campus Bio-Medico of Rome, Rome, Italy; 6 Multiple Sclerosis Center, Department of Neuroscience, City of Health and Science University Hospital, Turin, Italy; 7 Multiple Sclerosis Center, Neurology Unit, Ospedale Civile Di Ciriè, Turin, Italy; 8 Department of Translational Medicine, Neurology Unit, University of Piemonte Orientale, Novara, Italy; 9 Neurological Clinic, Marche Polytechnic University, Ancona, Italy; 10 Neurology and Neurorehabilitation Unit, IRCCS San Raffaele Hospital, Milan, Italy; 11 Vita-Salute San Raffaele University, 20132 Milan, Italy; 12 Neuroimaging Research Unit, IRCCS San Raffaele Hospital, 20132 Milan, Italy; 13 Neurophysiology Unit, IRCCS San Raffaele Hospital, 20132 Milan, Italy; 14 Department of Basic Medical Sciences, Neurosciences and Sense Organs, University of Bari “Aldo Moro, Bari, Italy; 15 Multiple Sclerosis Center, Neurology Unit and Stroke Unit, “Pugliese-Ciaccio” Hospital, Catanzaro, Italy; 16 IRCCS Institute of Neurological Science of Bologna, Bologna, Italy; 17 Department of Biomedical Science and Neuromotricity, University of Bologna, Bologna, Italy; 18 Second Division of Neurology, Department of Advanced Medical and Surgical Sciences, University of Campania “Luigi Vanvitelli”, Naples, Italy; 19 Multiple Sclerosis Clinical and Research Unit, Department of Systems Medicine, Tor Vergata University, Rome, Italy; 20 Multiple Sclerosis Center, Fabrizio Spaziani Hospital, Frosinone, Italy; 21 Department of Neurosciences, Centro di Ricerca per la Sclerosi Multipla (CERSM), Università Cattolica del Sacro Cuore, Rome, Italy; 22 Neurology Unit - Specialistic Department - ULSS5 , Polesana, Rovigo, Italy; 23 Multiple Sclerosis Centre, Neurology Unit and Stroke Unit, AOOR “Villa Sofia-Cervello, ” Palermo, Italy

**Keywords:** Multiple sclerosis, Primary progressive multiple sclerosis, Efficacy, Ocrelizumab

## Abstract

Ocrelizumab is a recombinant humanized monoclonal antibody selectively targeting CD20-expressing B cells. The effect of ocrelizumab on primary progressive multiple sclerosis (PPMS) has been evaluated during phase 3 trials that enrolled patients under 55 years with a maximum Expanded Disability Status Scale (EDSS) of 6.5. However, little is known on older disabled patients with longer disease duration. We aimed to assess the clinical effectiveness of ocrelizumab in PPMS patients out of the ORATORIO eligibility criteria. This multicenter retrospective study collected data about the effectiveness of ocrelizumab in PPMS patients who received treatment between May 2017 and June 2022 in the Italian MS centers contributing to the Italian MS Registry who adhered to the Compassionate Use Program. The confirmed EDSS worsening (CEW) (defined as either a ≥ 1-point or ≥ 2-point increase in EDSS score from baseline that was confirmed at T12 and T24) was calculated. At the date of data extraction, out of 887 PPMS patients who had received ocrelizumab, 589 (mean age 49.7 ± 10.7 years, 242 (41.1%) females) were enrolled. The mean follow-up period was 41.3 ± 12.3 months. A total of 149 (25.3%) received ocrelizumab according to the ORATORIO criteria (ORATORIO group) and 440 (74.7%) outside the ORATORIO criteria (non-ORATORIO group). No differences in terms of cumulative probabilities of 12 and 24 months of CEW of ≤ 1 point were found between ORATORIO and non-ORATORIO groups. Cox regression analyses showed that age older than 65 years (HR 2.51, 25% CI 1.07–3.65; *p* = 0.01) was associated with higher risk of CEW at 24 months. Patients not responding to ORATORIO criteria for reimbursability may benefit from ocrelizumab treatment, as disease activity, disease duration, and EDSS seem to not impact the disability outcome. Our results may suggest to extend the possible use of this powerful agent in selected patients under the age of 65 years.

**Supplementary Information:**

The online version contains supplementary material available at 10.1007/s13311-023-01415-y.

## Introduction

Multiple sclerosis (MS) is a chronic, highly complex, inflammatory, and degenerative demyelinating disease of the central nervous system (CNS) causing neurological deficits referable to damage to the spinal cord, brainstem, optic nerves, cerebellum, and cerebrum [1–3]. Primary progressive MS (PPMS) is a relatively rare form of MS, accounting for approximately 10–15% of MS patients and it is characterized by a progressive course from disease onset with or without superimposed discrete clinical attacks or relapses [4–6]. According to several studies, PPMS patients typically exhibit a disabling course from symptom onset with a higher proportion of patients presenting at onset with motor impairment, cerebellar ataxia, and brainstem symptoms than relapsing-onset patients [6–8]. According to a recent classification, progressive MS form is further categorized according to the presence of disease activity in “active” and “non-active” [9, 10]. Several treatments, including therapies approved for the treatment of relapsing forms of MS such as interferons, fingolimod, natalizumab, and alemtuzumab, demonstrated limited effect on reducing the disability progression in patients with PPMS [11, 12]. Currently, PPMS remains a high disabling condition with very high unmet medical need.

Ocrelizumab is a recombinant humanized monoclonal antibody that selectively targets CD20-expressing B cells. CD20 is expressed on the cell surface of the pre-B cells and mature and memory B cells but not on lymphoid stem cells and plasma cells. Ocrelizumab is able to selectively deplete CD20-expressing B cells, however, not affecting the B cell reconstitution and pre-existing humoral immunity, and preserving the innate immunity and the total T cell numbers [13, 14].

In the ORATORIO trial (Study WA25046), a randomized, double-blind, placebo-controlled clinical trial in patients with PPMS, patients treated with ocrelizumab showed a significant reduction in the risk of progression of clinical disability (measured by the Expanded Disability Status Scale [EDSS]) sustained for at least 12 weeks by 24% and for at least 24 weeks by 25% compared with placebo. In another analysis, 42.7% of patients treated with ocrelizumab had no evidence of progression compared to 29.1% of patients treated with placebo 120 [15].

Since 2017, in Italy, ocrelizumab has been provided under Compassionate Use Program (CUP) (MA30130) for subjects who have been diagnosed of PPMS fulfilling the inclusion criteria indicated by the protocol (diagnosis of PPMS according to Mc Donald criteria) [1, 16]. In January 2018, the European Medicines Agency (EMA) authorized ocrelizumab for the treatment of adult patients with relapsing MS showing clinical or imaging feature characteristic of disease activity and with early PPMS in terms of disease duration and disability level, and with active disease defined by clinical and MRI features [17].

However, PPMS patients treated with ocrelizumab under CUP not fulfilling the ORATORIO criteria but reporting benefits from the ocrelizumab therapy were allowed to continue the treatment in the Italian MA30130 program according to the clinical judgement.

The aim of this retrospective multicenter study was to assess clinical efficacy of ocrelizumab in a population of PPMS patients receiving this treatment under CUP not satisfying the ORATORIO eligibility criteria compared to those patients responding to the labeled criteria of the ORATORIO [15, 16].

## Methods

### Study Population

This multicenter retrospective study is based on prospectively collected data about the effectiveness of ocrelizumab in PPMS patients who received treatment between May 2017 and June 2022 in all Italian MS centers contributing to the Italian MS Registry. Data are collected from two populations of PPMS patients treated with ocrelizumab: 1. those patients who received ocrelizumab treatment in the CUP program and not responding to the ORATORIO criteria [1, 15, 16]. These data were obtained by the Italian participating centers to CUP and stored in a repository collecting general standard information of patients included in the MA30130 program [16]; 2. those patients who started their therapy under CUP and kept on being treated with ocrelizumab after its approval and according to the labeled criteria for PPMS [16]. Data of this group of patients have been extracted by a secondary repository which is the Italian MS Registry.

The study was approved by the Policlinico-Vittorio Emanuele (Catania, Italy; cod 54/2021/PO) Ethics Committee. Ethical committee approval was also obtained from each individual participating center.

The inclusion criteria were the following: adult age; ability to provide written informed consent and to be compliant with the requirements regarding the schedule of treatment and all related treatment procedures; diagnosis of PPMS in accordance with the revised McDonald criteria (2017) [3]; at least 4 treatment courses of ocrelizumab and at least 3 EDSS evaluations.

The ORATORIO eligibility criteria for ocrelizumab treatment included the following: an age of 18 to 55 years, a score on EDSS of 3.0 to 6.5, a disease duration less than 15 years in patients with an EDSS score of more than 5.0 at screening or less than 10 years in patients with an EDSS score of 5.0 or less [15]. According to the presence of ORATORIO eligibility criteria, patients were divided in ORATORIO and non-ORATORIO groups. Moreover, we further stratified patients in the non-ORATORIO group according to the age (≤ 55, 56–64 and ≥ 65 years), EDSS (≤ 6.5 and > 6.5), and disease duration (≤ 10–15 and > 10–15 years, according to the EDSS).

### Outcomes

In order to evaluate the disability progression during ocrelizumab treatment, EDSS evaluations were acquired at baseline (before ocrelizumab initiation), at 12 (T12) months, and 24 (T24) months after ocrelizumab initiation.

We stratified the cohort according to the presence of disease activity defined by the finding of active MS disease within the 24 months before starting ocrelizumab of clinical relapses and/or MRI activity [10]. Imaging features of inflammatory MRI activity were the following: at least one contrast-enhancing T1 lesion (CELs) or the development of at least 1 new or enlarging T2 lesions in comparison to the previous MRI [10].

The confirmed EDSS worsening (CEW) (defined as either a ≥ 1-point or ≥ 2-point increase in EDSS from baseline that was confirmed at T12 and T24) and the progression index (PI) (disability grade divided by duration of the disease) were calculated [18, 19].

### Statistical Analysis

Statistical analysis was carried out using the statistic package STATA 16.1. Normal distribution was tested by the Shapiro–Wilk test. Continuous variables were expressed by number of observations, mean, and standard deviation (SD). Categorical data were presented by absolute and relative frequencies (*n* and %) or contingency tables. In case of violation of the assumptions for *F* or *t*-tests, equivalent non-parametric statistics will be used. All demographical and clinical characteristics were compared between the two groups. The Spearman correlation coefficient (Rho) was used to evaluate the strength of correlations between the analyzed variables.

Analysis of variance (ANOVA) was also applied to test the main and interactive effects among different subgroups. The Bonferroni test was used to correct for multiple post hoc pairwise comparisons. Kaplan–Meier curves were used to estimate the cumulative risk of developing CEW of at least 1 point at 24 months (1-point CEW). The variables significantly (*p* < 0.15) related with time to CEW on univariate analysis were included in the multivariate model. Multivariable Cox proportional hazard models were used to identify demographic and clinical variables significantly and independently associated with the outcome (1-point CEW at 24 months). The Cox proportional hazard models were corrected for age, sex, disease duration, “active” MS disease (yes/no), and the number of ocrelizumab courses. The null hypothesis was rejected if *p* < 0.05 (also an indicator of statistical significance). The adjusted hazard ratios (HRs) and their 95% CI were used to interpret the final model. A two-sided *P* value of < 0.05 was considered statistically significant.

## Results

**Fig. 1:**
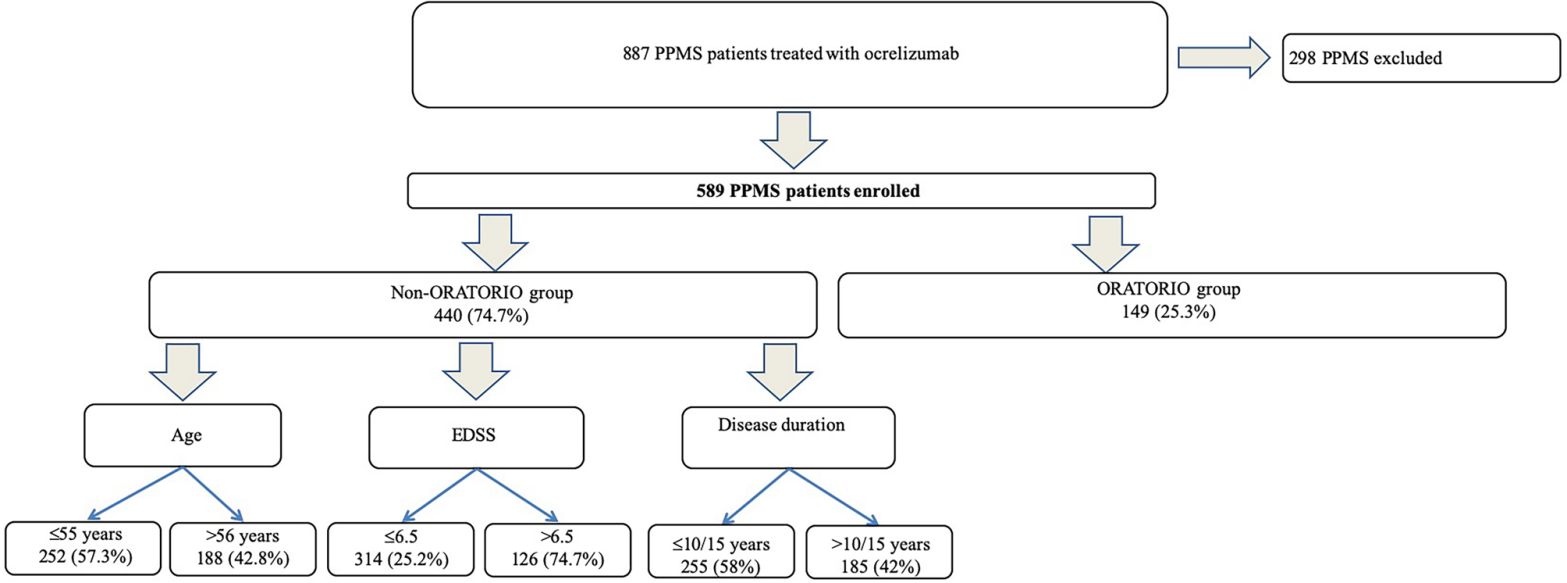
Patients’ selection flow chart. EDSS, Expanded Disability Status Scale; PPMS, primary progressive multiple sclerosis. The ORATORIO group includes patients fulfilling the ORATORIO eligibility criteria for ocrelizumab treatment (age of 18 to 55 years, EDSS of 3.0 to 6.5, disease duration less than 15 years in patients with an EDSS score of more than 5.0 at screening or less than 10 years in patients with an EDSS score of 5.0 or less [14]); non-ORATORIO includes patients not fulfilling the ORATORIO criteria

At the date of data extraction, out of 887 PPMS patients who had received ocrelizumab, 589 (mean age 49.7 ± 10.7 years, 242 [41.1%] females) fulfilled the inclusion criteria and were finally enrolled (Fig. 1). We found 149 (25.3%) received ocrelizumab according to the ORATORIO criteria (ORATORIO group) and 440 (74.7%) outside the ORATORIO criteria (non-ORATORIO group).

Among the patients in the non-ORATORIO group, 252 (57.3%) were older than 55 years, 126 (74.7%) had an EDSS higher than 6.5, and 185 (42%) had a disease duration longer than 10 or 15 years (according to EDSS) at the time of treatment initiation (Fig. 1).

The mean follow-up period was 41.3 ± 12.3 months. Demographic and clinical baseline characteristics are summarized in Table 1.

**Table 1:** Demographic and clinical characteristics of the two cohorts, ORATORIO and non-ORATORIO groups, according to the fulfillment of the ORATORIO criteria*

Tot. 589 *N* (%)	**ORATORIO group** 149 (25.3)	**Non-ORATORIO group** 440 (74.7)	***p* value**
Female; *N* (%)	48 (32.9)	194 (44.1)	0.1
Age (years); mean ± SD Median (range)	42.4 ± 7.9 41 (20–55)	52.1 ± 10.5 50 (23–77)	< 0.001
Age at onset (years); mean ± SD Median (range)	38.7 ± 9.5 37 (31–48)	39.4 ± 11.3 38 (35–51)	0.5
Disease duration (months); mean ± SD Median (range)	68.4 ± 43.2 65 (14–180)	157.2 ± 93.6 147 (14–444)	< 0.001
EDSS at diagnosis; mean ± SD Median (range)	3.5 ± 2.8 3 (2.0–4.5)	3.7 ± 2.9 3 (2.5–5.0)	0.9
EDSS at before starting OCR; mean ± SD Median (range)	5.4 ± 1.6 4.5 (3–5.5)	5.7 ± 1.7 5.5 (3.5–8.5)	0.8
EDSS at last follow up; mean ± SD Median (range)	5.8 ± 2.7 5 (4.5–6.5)	6.4 ± 2.2 6 (5.0–8.5)	0.6
No. of relapses before starting OCR; mean ± SD Median (range)	1.2 ± 1.6 1 (0–3)	1.2 ± 1.5 1 (0–3)	0.9
No. of relapses at last follow-up; mean ± SD Median (range)	1.3 ± 1.1 1 (1–2)	1.4 ± 1.2 1 (1–2)	0.9
No. of Gd-enhanced lesion before starting OCR; mean ± SD Median (range)	1.1 ± 1.2 (0–2)	1.0 ± 1.2 (0–2)	0.9
No. of Gd-enhanced lesion at last follow-up; mean ± SD Median (range)	0.6 ± 1.0 (0–2)	0.5 ± 0.9 (0–2)	0.7
No. of new or enlarged T2 lesion before starting OCR; mean ± SD Median (range)	1.5 ± 1.6 (0–3)	1.3 ± 1.8 (0–3)	0.6
No. of new or enlarged T2 lesion at last follow-up; mean ± SD Median (range)	1.8 ± 1.7 (0–5)	1.7 ± 1.8 (0–5)	0.6
Active disease** before starting OCR; *N* (%)	21 (14.1)	56 (12.7)	0.1
Progression index at 12 months; mean ± SD	0.80 ± 0.55	0.82 ± 0.68	0.8
Progression index at 24 months; mean ± SD	0.81 ± 0.75	0.83 ± 0.71	0.6
No. of OCR courses; mean ± SD Median (range)	6.4 ± 1.2 (1–5)	6.6 ± 1.8 (1–5)	0.7

*EDSS* Expanded Disability Status Scale, *OCR* ocrelizumab, *SD* standard deviation

^*^ORATORIO criteria: an age of 18 to 55 years, a score on EDSS of 3.0 to 6.5, a disease duration less than 15 years in patients with an EDSS score of more than 5.0 at screening or less than 10 years in patients with an EDSS score of 5.0 or less [15]

^**^Active disease was defined by the finding of clinical relapses and/or MRI activity within the 24 months before starting ocrelizumab [10]

The proportion of patients with disease activity at the time of ocrelizumab initiation was similar between ORATORIO and non-ORATORIO groups (Table 1). In addition, in the non-ORATORIO group, a higher percentage of active patients was found in those with EDSS > 6.5 (33 [23.2%] versus 23 [7.3%], *p* < 0.001), while no differences were found in the other subgroups (see Supplementary Materials).

The cumulative probabilities of 12 and 24 months of CEW of ≤ 1 point were 22.1% and 22.8%, respectively, in the ORATORIO group, and 18.4% and 27.5%, respectively, in the non-ORATORIO group. The cumulative probabilities of 12 and 24 months of CEW of ≥ 2 points were 3.4% and 5.4%, respectively, in the ORATORIO group, and 3.4% and 5%, respectively, in the non-ORATORIO group (Table 2).

**Table 2:** Differences in terms of confirmed disability worsening in ORATORIO and in non-ORATORIO groups

	**ORATORIO group** 149 (25.3)		**Non-ORATORIO group** 440 (74.7)		***p* value**
	*N*	%	*N*	%	
12 months confirmed worsening					
EDSS score ≥1.0	33	22.1	81	18.4	0.4
EDSS score ≥ 2.0	5	3.4	15	3.4	1.0
24 months confirmed worsening					
EDSS score ≥1.0	34	22.8	121	27.5	0.5
EDSS score ≥ 2.0	8	5.4	22	5	0.9

*EDSS* Expanded Disability Status Scale

Among patients in the non-ORATORIO group, patients with age > 56 years exhibited higher values of 12 and 24 months of CEW of ≥ 1 point and 12 and 24 months of CEW of ≥ 2 points compared to those patients aged < 55 years (Table 3).

**Table 3:** Differences in terms of confirmed disability worsening in patients stratified according to the age before starting ocrelizumab treatment

	**Age** ≤ **55 years** 252 (57.3)		**Age > 56 years** 188 (42.7)		***p* value**
	*N*	%	*N*	%	
12 months confirmed worsening					
EDSS score ≥ 1.0	24	9.5	37	19.7	0.01
EDSS score ≤ 2.0	3	1.2	6	3.2	0.2
24 months confirmed worsening					
EDSS score ≥ 1.0	46	18.3	57	30.3	0.02
EDSS score ≥ 2.0	4	1.6	9	4.8	0.06

*EDSS* Expanded Disability Status Scale

No significant differences in terms of 12 and 24 months of CEW of ≥ 1 point and 12 and 24 months of CEW of ≥ 2 points were found between patients stratified according to the EDSS and the disease duration at the time of ocrelizumab initiation (Tables 4 and 5).

**Table 4:** Differences in terms of confirmed disability worsening in patients stratified according to the EDSS before starting ocrelizumab treatment

	**EDSS** ≤ **6.5** 314 (25.2)		**EDSS > 6.5** 126 (74.7)		***p* value**
	*N*	%	*N*	%	
12 months confirmed worsening					
EDSS score ≥ 1.0	49	15.6	13	10.3	0.1
EDSS score ≥ 2.0	5	1.6	2	1.6	0.9
24 months confirmed worsening					
EDSS score ≥ 1.0	79	25.2	30	23.8	0.8
EDSS score ≥ 2.0	8	2.5	3	2.4	0.8

*EDSS* Expanded Disability Status Scale

**Table 5:** Differences in terms of confirmed disability worsening in patients stratified according to the disease duration before starting ocrelizumab treatment

Tot. 440	**Disease duration** ≤ **10/15 years** 255 (58)		**Disease duration > 10/15 years** 185 (42)		***p* value**
	*N*	*N*	*N*	%	
12 months confirmed worsening					
EDSS score ≥ 1.0	72	28.2	42	22.7	0.3
EDSS score ≥ 2.0	12	4.7	7	3.9	0.7
24 months confirmed worsening					
EDSS score ≥ 1.0	80	31.4	52	28.1	0.6
EDSS score ≥ 2.0	11	4.3	8	4.3	0.9

*EDSS* Expanded Disability Status Scale

Further stratification of patients aged > 55 years revealed that patients with age > 65 years at the time of ocrelizumab initiation showed significantly higher CEW of ≥ 1 point at 12 months and of CEW of ≥ 2 points at 12 and 24 months compared to those patients with age between 56 and 64 years and to those with age ≤ 55 years (Table 6).

**Table 6:** Differences in terms of confirmed disability worsening in patients stratified according to the age ≤ 55 years, 56–64 years, and > 65 years before starting ocrelizumab treatment

Tot. 440	**Age ≤ 55 years** 252 (57.3) (A)		**Age 56–64 years** 149 (33.9) (B)		**Age ≥ 65 years** 39 (8.9) (C)		***p* value**	**ANOVA after Bonferroni correction**
	*N*	%	*N*	%	*N*	%		
12 months confirmed worsening								
EDSS score ≥ 1.0	24	9.5	20	13.4	17	43.6	**0.001**	A vs B 0.3; B vs C < 0.001; A vs C < 0.001
EDSS score ≥ 2.0	3	1.2	4	2.7	2	5.1	0.2	
24 months confirmed worsening								
EDSS score ≥ 1.0	46	18.3	32	21.5	25	64.1	**0.001**	A vs B 0.5; B vs C < 0.001
EDSS score ≥ 2.0	4	1.6	5	3.4	4	10.3	**0.05**	A vs C < 0.001; A vs B 0.3; B vs C 0.09; A vs C 0.003

Bold indicates the *p* values statistically significant. *EDSS* Expanded Disability Status Scale

**Fig. 2:**
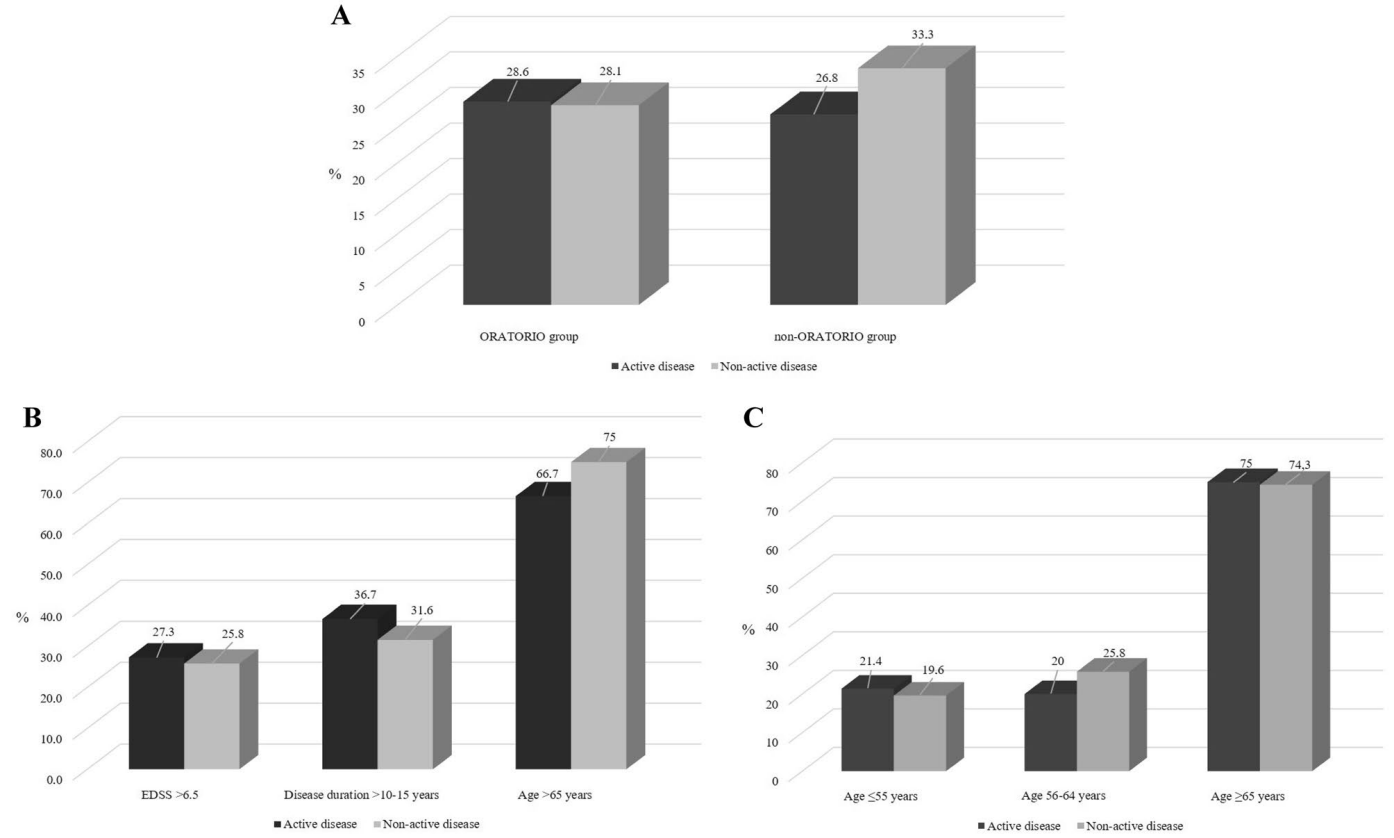
Proportion of patients who reached 1-point CEW at 24 months, stratified according to the presence of disease activity in ORATORIO and non-ORATORIO groups (**A**), in each non-ORATORIO subgroup (**B**), and in each age category (**C**). CEW, confirmed EDSS worsening of at least 1 point at 24 months; EDSS, Expanded Disability Status Scale

No significant differences in terms of 1-point CEW at 24 months were found between patients stratified according to the presence of disease activity at the time of ocrelizumab initiation (*p* = 0.8 and *p* = 0.5 in ORATORIO and non-ORATORIO groups, respectively) (Fig. 2A). Particularly, in the non-ORATORIO group, proportion of patients who reached 1-point CEW at 24 months was similar between “active” and “non-active,” in each subgroup (Fig. 2B). Stratifying the age at the time of ocrelizumab initiation in three categories (≤ 55, 56–64, and ≥ 65 years), no significant differences were found in 1-point CEW at 24 months (Fig. 2C).

In addition, no differences in terms of PI at 12 and 24 months were found between the ORATORIO and non-ORATORIO group (Table 1).

**Fig. 3:**
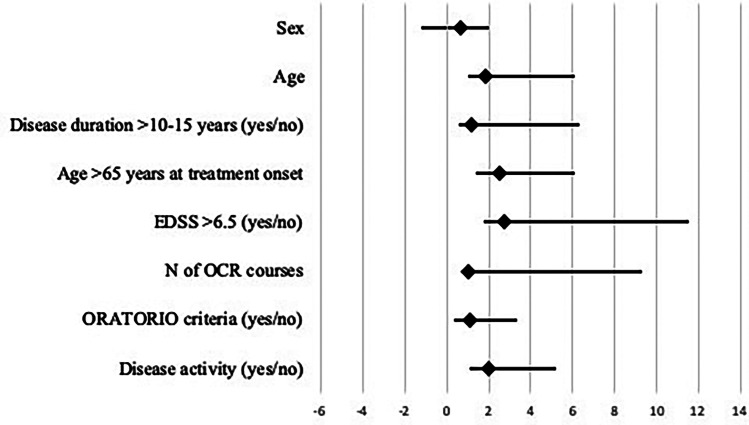
Cox regression analysis of developing confirmed EDSS worsening (CEW) at 24 months. EDSS, Expanded Disability Status Scale; OCR, ocrelizumab.*p value = 0.01

**Fig. 4:**
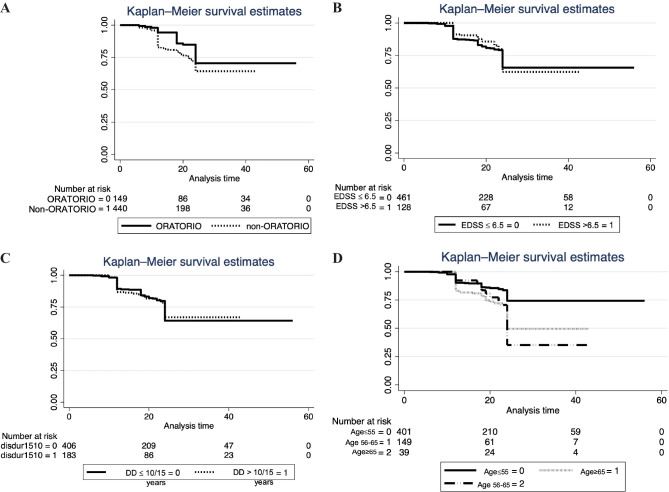
Kaplan–Meier curves for the time of reaching 1-point CEW at 24 months during treatment with ocrelizumab in the entire cohort (**A**), and in patients stratified according to EDSS (≤ 6.5 and > 6.5) (**B**), disease duration (≤ 15 or 10 and > 15 or 10 years) (**C**), and age (≤ 55, 56–64 and > 65 years) (**D**). CEW, confirmed EDSS worsening of at least 1 point at 24 months; EDSS, Expanded Disability Status Scale; DD, disease duration

The Cox proportional hazard model showed that age older than 65 years at the time of ocrelizumab initiation was independently associated with higher risk of CEW at 24 months (HR 2.51, 25% CI 1.07–3.65; *p* = 0.01). The results of the Cox regression analysis for time to CEW in all patients are illustrated in Fig. 3. The Kaplan–Meier–estimated cumulative risk of CEW was similar in ORATORIO and non-ORATORIO groups (*p* = 0.6) (Fig. 4A) and stratifying according to EDSS (*p* = 0.8) (Fig. 4B) and to disease duration (*p* = 0.8) (Fig. 4C). Age older than 65 years was significantly associated with a shorter time to reach CEW (*p* < 0.01) (Fig. 4D).

## Discussion

In this multicenter, observational study, ocrelizumab administrations showed similar effects on disability progression in both PPMS patients responding or not to the ORATORIO eligibility criteria.

Among patients who did not present the ORATORIO criteria, those with age > 65 years at the time of ocrelizumab initiation showed significantly higher CEW of ≥ 1 point at 12 months and of CEW of ≥ 2 points at 12 and 24 months compared to those patients with age between 56 and 64 years and with age ≤ 55 years. No significant differences in terms of 12 and 24 months of CEW of ≤ 1 point and 12 and 24 months of CEW of ≥ 2 points were found between patients stratified according to the EDSS and the disease duration at the time of ocrelizumab initiation.

The anti-CD20 monoclonal antibody ocrelizumab was the first disease-modifying therapy to be approved for treatment of PPMS. A previous phase 2–3 trial of rituximab (OLYMPUS), a chimeric monoclonal anti-CD20 antibody, in PPMS patients did not meet its primary efficacy endpoint; however, a subgroup analysis demonstrated that patients with age < 51 years showed delayed progression of disability [20]. Subsequently, the ORATORIO trial investigated the effect of ocrelizumab in patients’ PPMS and randomized 732 patients to treatment with either ocrelizumab or placebo (2:1) for 3 years. In this study, ocrelizumab was able to reduce the percentage of patients with 12-week confirmed EDSS disability progression (HR = 0.76; 95% CI, 0.59–0.98; *p* < 0.03) [15].

Later, real-world evidences have confirmed the effectiveness of ocrelizumab in reducing relapses, MRI activity, and slowing down the rate of progression in PPMS patients [21, 22], although another recent observational comparative study of a small cohort of 13 PPMS and 29 relapsing–remitting MS indicated that the effect of ocrelizumab on disability progression was more evident for relapsing–remitting MS [23].

In line with our results showing that patients older than 55 years had similar disability progression compared to younger ones, a recent retrospective real-world study demonstrated that among 56 patients older than the age of 55 at the time of ocrelizumab initiation, a high percentage of patients, about 60%, remained stable or improved after 2 years of ocrelizumab treatment [24].

On the other hand, we found that age older than 65 is associated to higher risk of disability progression. Indeed, age-related functional changes of the innate and adaptive immune, referred to as immunosenescence, with the resulting low-grade proinflammatory state (inflammaging), may impact the efficacy as well as the safety profile of current DMTs [25]. The immunosenescence can affect the T and B cells, monocytes and macrophages, microglia, dendritic cells, and natural killer cells, also inducing a reduction and functional alterations of the naïve B cell population of clonal expansion capabilities of memory cells and of antibody levels and antibody specificity [26, 27].

Moreover, several studies observed that old age in MS is associated to a greater multimorbidity risk and, in turn, the presence of comorbidities, such as cardiovascular diseases, is considered a risk factor for disability accumulation [28, 29]. Age-associated comorbidities may also influence the risk–benefit analysis for DMTs and be accompanied to reduced efficacy.

A recent meta-analysis of randomized, blinded clinical trials of MS DMTs against placebo or active comparator involving more than 28,000 MS patients revealed that the efficacy of immunomodulatory DMTs on disability progression strongly decreases with age, demonstrating a loss of efficacy at an average age of 53 [30]. Particularly, highly active drugs seemed to reduce their higher efficacy, as compared to low-efficacy drugs in patients aged 40.5 years and older [30].

Notably, current clinical trials have excluded patients over age 55, and thus, there are no data suggesting that DMTs are either effective in the elderly, especially in those without disease activity. Indeed, an age gap exists between the MS clinical trial and real-world populations due to the growing numbers of elderly people with MS [31]. This makes clinical trial results less applicable to the aging real-world MS population in terms of age and age-related changes in disease activity.

In our study, disease duration longer than 10–15 years and EDSS < 6.5 seemed to not affect the disability outcome. The evidence regarding the predictive power of disease duration on disease progression was mixed, and several studies have shown that disease duration did not consistently predict disability worsening, particularly in the long-term [32, 33].

Similarly, the prognostic limitations of the EDSS across several domains has been widely demonstrated [34]. Indeed, while lower EDSS values are mainly based on impairments detected by the neurologic examination, values higher than 4 are deeply influenced by walking disability. In addition, several studies suggested that EDSS scores of 6 and higher are less sensitive to change in progression of the disease [35, 36]. Furthermore, it should be noted that EDSS does not adequately gather the possible changes of cognitive function, upper extremity ability, and fatigue, which are demonstrated to be relevant predictors of long-term disease progression in MS [33].

In addition, as observed in a post hoc analysis of OPERA I and II trials on relapsing MS patients, sex did not affect progression outcomes in our cohort of patients [37]. On the contrary, another real-world study showed that male gender and longer follow-up period were independent predictors for disability progression in a cohort of 48 PPMS with a follow-up longer than 1 year [38].

Interestingly, the presence of disease activity at the time of treatment initiation seemed to not influence the outcome. This is apparently in contrast with the current literature showing that powerful therapies are more efficacious in reducing relapses in patients with active progressive MS [39]. However, subsequent analyses on ORATORIO dataset could not demonstrate significant differences regarding the response to ocrelizumab in active and non-active PPMS patients [14, 40]. Indeed, our current understanding of disease activity mainly focuses on inflammation-related relapses and/or MRI activity [10, 14], while to date, the effects of powerful DMTs on disability accumulation without immunological activity (also called “progression independent of relapse activity” [PIRA]) are less well known [41].

Our study has several limits. The retrospective design may have limited the statistical power of our results. Moreover, the use of EDSS as clinical endpoint may underestimate the possible worsening of disability progression in our cohort because of a low event rate and fluctuation in scores. Finally, our study did not investigate the safety profile of ocrelizumab in patients who did not fulfill the ORATORIO criteria; further analyses are required to characterize the risk of adverse events, including progressive multifocal leukoencephalopathy (PML) in this group of patients, particularly in the elderly. As the probability of active disease declines with age while the susceptibility to adverse events increases, the risks versus benefits of using ocrelizumab in the elderly should be verified in longitudinal studies.

In conclusion, our results showed that disease activity, disease duration, and EDSS at the time of ocrelizumab initiation seem to not impact the disability outcomes. Patients not responding to ORATORIO criteria for reimbursability may benefit from ocrelizumab treatment, thus suggesting to extend the possible use of this powerful agent in selected patients under the age of 65 years.

### Supplementary Information

Below is the link to the electronic supplementary material.

Supplementary file1 (DOCX 31 KB)

## Data Availability

Dataset is available under reasonable request to the corresponding author.
