# Supplementary material for: Effectiveness of Ocrelizumab in Primary Progressive Multiple Sclerosis: a Multicenter, Retrospective, Real-world Study (OPPORTUNITY)
Source: Neurotherapeutics. 2023 Aug 23;20(6):1696–706. doi: 10.1007/s13311-023-01415-y (PMC10684838; doi:10.1007/s13311-023-01415-y)
Supplement: Supplementary file 1 — Supplementary file1 (DOCX 31 KB) [file 13311_2023_1415_MOESM1_ESM.docx]

**Tables and figures**

Table 1: Demographic and clinical characteristics of the two cohorts, ORATORIO and non-ORATORIO groups according to the fulfillment of the ORATORIO criteria*.

| Tot. 589  N (%) | **ORATORIO**  **group**  149 (25.3) | **Non-ORATORIO**  **group**  440 (74.7) | **P value** |
| --- | --- | --- | --- |
| *Female; N (%)* | 48 (32.9) | 194 (44.1) | 0.1 |
| *Age (years); mean ±SD*  *Median (range)* | 42.4 ± 7.9  41 (20-55) | 52.1 ± 10.5  50 (23-77) | <0.001 |
| *Age at onset (years); mean±SD*  *Median (range)* | 38.7 ± 9.5  37 (31-48) | 39.4 ± 11.3  38 (35-51) | <0.001 |
| *Disease duration (months); mean±SD*  *Median (range)* | 68.4 ± 43.2  65 (14-180) | 157.2 ± 93.6  147 (14-444) | <0.001 |
| *EDSS at diagnosis; mean±SD*  *Median (range)* | 3.5 ± 2.8  3 (2.0-4.5) | 3.7 ± 2.9  3 (2.5-5.0) | 0.9 |
| *EDSS at before starting OCR;mean±SD*  *Median (range)* | 5.4 ± 1.6  4.5 (3-5.5) | 5.7 ± 1.7  5.5 (3.5-8.5) | 0.8 |
| *EDSS at last follow up; mean±SD*  *Median (range)* | 5.8 ± 2.7  5 (4.5-6.5) | 6.4 ± 2.2  6 (5.0-8.5) | 0.6 |
| *N. of relapses before starting OCR; mean±SD*  *Median (range)* | 1.2 ± 1.6  1 (0-3) | 1.2 ± 1.5  1 (0-3) | 0.9 |
| *N. of relapses at last follow-up; mean±SD*  *Median (range)* | 1.3 ± 1.1  1 (1-2) | 1.4 ± 1.2  1 (1-2) | 0.9 |
| *N. of Gd-enhanced lesion before starting OCR; mean±SD*  *Median (range)* | 1.1 ± 1.2  (0-2) | 1. ± 1.2   (0-2) | 0.9 |
| *N. of Gd-enhanced lesion at last follow-up; mean±SD*  *Median (range)* | 0.6 ± 1.0  (0-2) | 0.5 ± 0.9  (0-2) | 0.7 |
| *N. of new or enlarged T2 lesion before starting OCR; mean±SD*  *Median (range)* | 1.5 ± 1.6  (0-3) | 1.3 ± 1.8  (0-3) | 0.6 |
| *N. of new or enlarged T2 lesion at last follow-up; mean±SD*  *Median (range)* | 1.8 ± 1.7  (0-5) | 1.7 ± 1.8  (0-5) | 0.6 |
| ***Active disease** before starting OCR; N (%)*** | 21 (14.1) | 56 (12.7) | 0.1 |
| *Progression index at 12 months; mean±SD* | 0.80 ± 0.55 | 0.82 ± 0.68 | 0.8 |
| *Progression index at 24 months; mean±SD* | 0.81± 0.75 | 0.83 ± 0.71 | 0.6 |
| *N of OCR courses; mean±SD*  *Median (range)* | 6.4 ± 1.2  (1-5) | 6.6 ± 1.8  (1-5) | 0.7 |

EDSS: Expanded Disability Status Scale; OCR: ocrelizumab; SD; standard deviation.

*ORATORIO criteria: an age of 18 to 55 years, a score on EDSS of 3.0 to 6.5, a disease duration less than 15 years in patients with an EDSS score of more than 5.0 at screening or less than 10 years in patients with an EDSS score of 5.0 or less [15].

** Active disease was defined by the finding of clinical relapses and/or MRI activity within the 24 months before starting ocrelizumab (10).

Table 2. Differences in terms of confirmed disability worsening in ORATORIO and in non-ORATORIO groups.

|  | **ORATORIO**  **group**  149 (25.3) | | **Non-ORATORIO**  **group**  440 (74.7) | | **P value** |
| --- | --- | --- | --- | --- | --- |
|  | N | % | N | % |  |
| *12 months confirmed worsening*  *EDSS score ≤1.0*  *EDSS score* ≥*2.0* | 33  5 | 22.1  3.4 | 81  15 | 18.4  3.4 | 0.4  1.0 |
| *24 months confirmed worsening*  *EDSS score ≤1.0*  *EDSS score* ≥*2.0* | 34  8 | 22.8  5.4 | 121  22 | 27.5  5 | 0.5  0.9 |

EDSS: Expanded Disability Status Scale.

Table 3. Differences in terms of confirmed disability worsening in patients stratified according to the age before starting ocrelizumab treatment.

|  | **Age**  **≤55 years**  252 (57.3) | | **Age**  **>56 years**  188 (42.7) | | **P value** |
| --- | --- | --- | --- | --- | --- |
|  | N | % | N | % |  |
| *12 months confirmed worsening*  *EDSS score ≤1.0*  *EDSS score* ≥*2.0* | 24  3 | 9.5  1.2 | 37  6 | 19.7  3.2 | 0.01  0.2 |
| *24 months confirmed worsening*  *EDSS score ≤1.0*  *EDSS score* ≥*2.0* | 46  4 | 18.3  1.6 | 57  9 | 30.3  4.8 | 0.02  0.06 |

EDSS: Expanded Disability Status Scale.

Table 4. Differences in terms of confirmed disability worsening in patients stratified according to the EDSS before starting ocrelizumab treatment.

|  | **EDSS**  **≤6.5**  314 (25.2) | | **EDSS**  **>6.5**  126 (74.7) | | **P value** |
| --- | --- | --- | --- | --- | --- |
|  | N | % | N | % |  |
| *12 months confirmed worsening*  *EDSS score ≤1.0*  *EDSS score* ≥*2.0* | 49  5 | 15.6  1.6 | 13  2 | 10.3  1.6 | 0.1  0.9 |
| *24 months confirmed worsening*  *EDSS score ≤1.0*  *EDSS score* ≥*2.0* | 79  8 | 25.2  2.5 | 30  3 | 23.8  2.4 | 0.8  0.8 |

EDSS: Expanded Disability Status Scale.

Table 5. Differences in terms of confirmed disability worsening in patients stratified according to the disease duration before starting ocrelizumab treatment.

| Tot. 440 | **Disease**  **Duration**  ≤**10/15 years**  255 (58) | | **Disease**  **Duration**  **>10/15 years**  185 (42) | | **P value** |
| --- | --- | --- | --- | --- | --- |
|  | N | N | N | % |  |
| *12 months confirmed worsening*  *EDSS score ≤1.0*  *EDSS score* ≥*2.0* | 72  12 | 28.2  4.7 | 42  7 | 22.7  3.9 | 0.3  0.7 |
| *24 months confirmed worsening*  *EDSS score ≤1.0*  *EDSS score* ≥*2.0* | 80  11 | 31.4  4.3 | 52  8 | 28.1  4.3 | 0.6  0.9 |

EDSS: Expanded Disability Status Scale.

Table 6. Differences in terms of confirmed disability worsening in patients stratified according to the age ≤55 years, 56-64 years and >65 years before starting ocrelizumab treatment.

| Tot. 440 | **Age**  **≤55 years**  252 (57.3)  **(A)** | | **Age**  **56-64 years**  149 (33.9)  (B) | | **Age**  **≥65 years**  39 (8.9)  (C) | | **P value** | **ANOVA after Bonferroni**  **correction** |
| --- | --- | --- | --- | --- | --- | --- | --- | --- |
|  | N | % | N | % | N | % |  |  |
| *12 months confirmed worsening*  *EDSS score ≤1.0*  *EDSS score* ≥*2.0* | 24  3 | 9.5  1.2 | 20  4 | 13.4  2.7 | 17  2 | 43.6  5.1 | **0.001**  0.2 | A vs B 0.3; B vs C <0.001; A vs C <0.001; |
| *24 months confirmed worsening*  *EDSS score ≤1.0*  *EDSS score* ≥*2.0* | 46  4 | 18.3  1.6 | 32  5 | 21.5  3.4 | 25  4 | 64.1  10.3 | **0.001**  **0.05** | A vs B 0.5; B vs C <0.001; A vs C <0.001  A vs B 0.3; B vs C 0.09; A vs C 0.003 |

EDSS: Expanded Disability Status Scale.

Supplementary Material. Proportion of patients with disease activity in the non-ORATORIO group, stratified according to the age, EDSS and disease duration before starting ocrelizumab treatment.

| N (%) | **EDSS**  **≤6.5**  314 (25.2) | **EDSS**  **>6.5**  126 (74.7) | **p value** | **Disease**  **Duration**  ≤**10/15 years**  255  (58) | **Disease**  **Duration**  **>10/15 years**  185  (42) | **p value** | **Age**  ≤**55 years**  252 (57.3) | **Age**  **56-64 years**  149 (33.9) | **Age**  **≥65 years**  39 (8.9) | **p value** |
| --- | --- | --- | --- | --- | --- | --- | --- | --- | --- | --- |
| *‘Active’ disease; N*  *(%)* | 23  (7.3) | 33  (26.2) | 0.0001 | 26  (10.2) | 30  (16.2) | 0.1 | 28  (11.1) | 25  (16.8) | 4  (28.2) | 0.3 |

EDSS: Expanded Disability Status Scale.

Figure 1. Patients’ selection flow chart.

EDSS: expanded disability status scale; PPMS: primary progressive multiple sclerosis.

ORATORIO group includes patients fulfilling the ORATORIO eligibility criteria for ocrelizumab treatment (age of 18 to 55 years, EDSS of 3.0 to 6.5, disease duration less than 15 years in patients with an EDSS score of more than 5.0 at screening or less than 10 years in patients with an EDSS score of 5.0 or less (14)); non-ORATORIO includes patients not fulfilling the ORATORIO criteria.

Figure 2. Proportion of patients who reached 1-point CEW at 24 months, stratified according to the presence of disease activity, in ORATORIO and non-ORATORIO groups (A), in each non-ORATORIO subgroup (B), and in each age category (C).

CEW: confirmed EDSS worsening of at least 1 point at 24months; EDSS: Expanded Disability Status Scale

Figure 3. Cox regression analysis of developing confirmed EDSS worsening (CEW) at 24 months.

EDSS: Expanded Disability Status Scale; OCR: ocrelizumab.

* p value=0.01

Figure 4. Kaplan Meier curves for the time of reaching 1-point CEW at 24 months during treatment with ocrelizumab in the entire cohort (A), and in patients stratified according to EDSS (≤ 6.5 and >6.5) (B), disease duration (≤ 15 or 10 and > 15 0r 10 years) (C), and age (≤ 55, 56-64 and >65 years) (D).

CEW: confirmed EDSS worsening of at least 1 point at 24months; EDSS: Expanded Disability Status Scale; DD: disease duration.
